# Developing excellence in biostatistics leadership, training and science in Africa: How the Sub-Saharan Africa Consortium for Advanced Biostatistics (SSACAB) training unites expertise to deliver excellence

**DOI:** 10.12688/aasopenres.13144.2

**Published:** 2020-12-22

**Authors:** Tobias F. Chirwa, Zvifadzo Matsena Zingoni, Pascalia Munyewende, Samuel O. Manda, Henry Mwambi, Ngianga-Bakwin Kandala, Samson Kinyanjui, Taryn Young, Eustasius Musenge, Jupiter Simbeye, Patrick Musonda, Michael Johnson Mahande, Patrick Weke, Nelson Owuor Onyango, Lawrence Kazembe, Nazarius Mbona Tumwesigye, Khangelani Zuma, Nonhlanhla Yende-Zuma, Marie-Claire Omanyondo Ohambe, Emmanuel Nakua Kweku, Innocent Maposa, Birhanu Ayele, Thomas Achia, Rhoderick Machekano, Lehana Thabane, Jonathan Levin, Marinus J.C. Eijkemans, James Carpenter, Charles Chasela, Kerstin Klipstein-Grobusch, Jim Todd

**Affiliations:** 1 Division of Epidemiology and Biostatistics, School of Public Health, University of the Witwatersrand, Johannesburg, 2193, South Africa; 2 Department of Statistics, University of Pretoria, Pretoria, South Africa; 3 School of Mathematics, Statistics & Computer Science, University of KwaZulu Natal, Pietermaritzburg, South Africa; 4 Biostatistics Research Unit, South African Medical Research Council, Pretoria, South Africa; 5 Research, Kenya Medical Research Institute-Wellcome Trust Research Programme, Kilifi, Kenya; 6 Division of Epidemiology and Biostatistics, Department of Global Health, Stellenbosch University, Cape Town, South Africa; 7 Mathematical Sciences Department, Chancellor College, University of Malawi, Zomba, Malawi; 8 Department of Epidemiology and Biostatistics, School of Medicine, University of Zambia, Lusaka, Zambia; 9 Department of Epidemiology and Biostatistics, Institute of Public Health, Kilimanjaro Christian Medical University College, Moshi, Tanzania; 10 School of Mathematics, College of Biological and Physical Science, University of Nairobi, Nairobi, Kenya; 11 Department of Statistics and Population Studies,, University of Namibia, Windhoek, Namibia; 12 Department of Epidemiology and Biostatistics, Makerere University, Kampala, Uganda; 13 Human and Social Capabilities (HSC) Research Division, Human Sciences Research Council, Pretoria, South Africa; 14 Statistics and Data Management, Centre for the AIDS Programme of Research in South Africa (CAPRISA), Durban, South Africa; 15 Biostatistics, Doctoral School, Institut Superieur Des Techniques Medicales De Kinshasa (ISTM), Kinshasa, Democratic Republic of the Congo; 16 Department of Epidemiology and Biostatistics, Kwame Nkrumah University of Science and Technology (KNUST), Kumasi, Ghana; 17 Division of Global HIV & TB (DGHT), United States Centers for Disease Control and Prevention, KEMRI Campus, Kisumu, Kenya; 18 Department of Health Research Methods, Evidence, and Impact, McMaster University, Hamilton, Canada; 19 Julius Center for Health Sciences and Primary Care, University Medical Center Utrecht, Utrecht University, Utrecht, The Netherlands; 20 Department of Population Health, London School of Hygiene and Tropical Medicine, London, UK; 21 Research statistics, Right to Care, Pretoria, South Africa

**Keywords:** biostatistics, capacity building, DELTAS, SSACAB, programme achievements, networks and partnerships, sub-Saharan Africa

## Abstract

The increase in health research in sub-Saharan Africa (SSA) has led to a high demand for biostatisticians to develop study designs, contribute and apply statistical methods in data analyses. Initiatives exist to address the dearth in statistical capacity and lack of local biostatisticians in SSA health projects. The Sub-Saharan African Consortium for Advanced Biostatistics (SSACAB) led by African institutions was initiated to improve biostatistical capacity according to the needs identified by African institutions, through collaborative masters and doctoral training in biostatistics. SACCAB has created a critical mass of biostatisticians and a network of institutions over the last five years and has strengthened biostatistics resources and capacity for health research studies in SSA.  SSACAB comprises 11 universities and four research institutions which are supported by four European universities.  In 2015, only four universities had established Masters programmes in biostatistics and SSACAB supported the remaining seven to develop Masters programmes. In 2019 the University of the Witwatersrand became the first African institution to gain Royal Statistical Society accreditation for a Biostatistics Masters programme. A total of 150 fellows have been awarded scholarships to date of which 123 are Masters fellowships (41 female) of whom 58 have already graduated. Graduates have been employed in African academic (19) and research (15) institutions and 10 have enrolled for PhD studies. A total of 27 (10 female) PhD fellowships have been awarded; 4 of them are due to graduate by 2020. To date, SSACAB Masters and PhD students have published 17 and 31 peer-reviewed articles, respectively. SSACAB has also facilitated well-attended conferences, face-to-face and online short courses. Pooling of limited biostatistics resources in SSA combined with co-funding from external partners has shown to be an effective strategy for the development and teaching of advanced biostatistics methods, supervision and mentoring of PhD candidates.

## Disclaimer

The views expressed in this article are those of the author(s). Publication in AAS Open Research does not imply endorsement by the AAS.

## Introduction

Biomedical research plays a key role in strengthening health systems, identifying and addressing health needs, and in improving health through building a local evidence base which helps to inform policy and practice ( Agnandji *et al.*, 2012; Franzen *et al.,* 2017). The data generation from donor-funded health sciences research initiatives has increased in the past decade, which has been matched by increased governmental funding for healthcare from most African countries ( Gezmu *et al.*, 2011). However, in sub-Saharan Africa (SSA) there is a dearth in statistical capacity to analyse the vast amounts of research and routinely collected patient data ( Cole *et al.*, 2014; Thomson *et al.*, 2016). Biostatisticians (biomedical methodologists) in the region are required in particular at universities, research institutions, governmental institutions, industrial settings and pharmaceutical companies; hence, there is a great demand for well-trained biostatisticians. However, there has been limited support to enhance the expansion of biostatistics at tertiary institutions. Moreover, the current pool of biostatisticians is too small to sufficiently provide the much needed statistical support and lead statistical research/methodological development in SSA ( Gezmu *et al.*, 2011; Machekano *et al.*, 2015). 

With the increasing availability of data resources such as routinely collected health data and publicly available data in addition to the increased focus in data science to guide evidence-based policies, an increased mass of biostatisticians is needed to analyse these data ( Fegan *et al.,* 2011). Training biostatisticians abroad is expensive, and many biostatisticians who undertake advanced training in foreign countries do not return. However, to date, in SSA the number of institutions offering biostatistics programs to build the critical mass to fill the urgent need for biostatisticians is limited, especially for post-graduate training ( Esterhuizen *et al.,* 2019; Machekano *et al.*, 2016; Thomson *et al.*, 2016). Furthermore, the statistics departments offering such training need to be linked to research institutions to ensure students have a practical understanding of the clinical and scientific context of the data they analyse ( Turner *et al.*, 2016) and to provide job opportunities and career pathways for their graduates.

As much as the Sub-Saharan Consortium of Advanced Biostatistics (SSACAB) was established to empower biostatisticians in this era of evidence-based health management and policy formulation, the consortium complements other currently existing initiatives that offer training in mathematics, biology, physics, economics, statistics, and epidemiology but in a much more structured and integrated manner. Several initiatives exist in SSA, but most of these initiatives focus on capacity building in disciplines other than biostatistics ( Ezeh *et al.*, 2010). Moreover, these initiatives rely on biostatisticians to fully meet their deliverables which shows the importance of biostatisticians in the current times. Some of these initiatives are highlighted in Box 1.

**Box 1. Examples of the research capacity initiatives in sub-Saharan Africa**

- Mathematics in South Africa (MASAMU) Program at Auburn University (funded by the National Science Foundation (NSF)) ( https://www.masamu.auburn.edu/). The MASAMU overall objective is to enhance research in the mathematical sciences within the Southern Africa Mathematical Sciences Association (SAMSA) institutions;
- African Institute for Mathematical Sciences (AIMS) with six centres of excellence across Africa, in Ghana, Cameroon, Senegal, Tanzania, and Rwanda, and South Africa ( https://aims.ac.za/);
- South African Centre for Epidemiological Modelling and Analysis (SACEMA) ( http://www.sacema.org/);
- Consortium of Advanced Research Training in Africa (CARTA) ( http://cartafrica.org/);
- Regional initiatives include Training Health Researchers into Vocational Excellence in East Africa (THRiVE) program; https://thrive.or.ug/
- Netherlands–African Partnership for Capacity Development and Clinical Interventions of Poverty-related Diseases (NACCAP) which is a programme nested under the Dutch Research Council (NWO) ( https://www.nwo.nl/en);
- Health Research Capacity Strengthening Initiative in Malawi (HRCSR) partnership between the U.K. Department for International Development (DFID) ( Liverpool Associates in Tropical Health, 2010); and the
- International Development and Research Centre (IDRC) Canada ( https://www.idrc.ca/en) and the Wellcome Trust ( https://wellcome.ac.uk/)

## Pooling of limited resources for advanced biostatistics training

North-South collaboration between high-income countries and low-and middle-income countries (LMIC) can help to transfer knowledge and skills to develop biostatistical capacity, retain skilled graduates, and increase research output ( Franzen *et al.,* 2017; Kellerman *et al.*, 2012; Nachega *et al.*, 2012; Uthman *et al.*, 2015). Since 2010, regional meetings have explored ways to improve South-South collaboration in biostatistics to pool resources and build training capacity ( Machekano *et al.*, 2015). The funding of the SSACAB programme by the Wellcome Trust under the Developing Excellence in Leadership, Training and Science in Africa Scheme (DELTAS) provided the opportunity to initiate South-South collaborations in biostatistics training and ensured a well-coordinated advanced training in biostatistics. This was done to build a critical mass for research and biostatistics leadership. The SSACAB programme aims to develop and improve skills among health researchers and academics in Africa as well as grow the biostatistics discipline in the region through Masters and PhD level training. SSACAB aims to create nodes of biostatistical excellence, which train public health researchers with advanced skills and expertise in biostatistics; and to provide a sustainable career path for African statisticians.

## The Sub-Saharan Africa Consortium for Advanced Biostatistics programme

**Figure 1.**
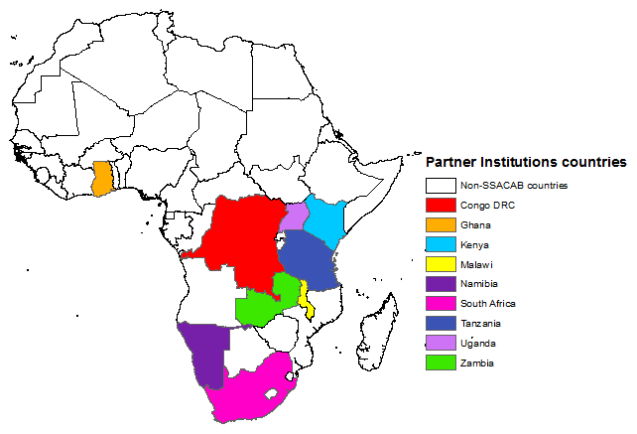
The distribution of the Sub-Saharan Africa Consortium for Advanced Biostatistics (SSACAB) partner institutions.

SSACAB comprises 11 African universities in nine countries ( Figure 1 top map) with interest in developing biostatistics degrees, four research institutions and four Northern partners.

The SSACAB (see Table 1 for a full list of SSACAB partners) aims to address three major objectives:

1. Develop, strengthen and implement high-quality biostatistics Masters level training
2. Provide PhD level training to develop expertise, skills, and become research leaders in biostatistics in Africa; and
3. Build a sustainable network of biostatisticians and statistically informed researchers within each country through outreach, mentoring and transferring skills, workshops and conferences.

**Table 1.** List of the Sub-Saharan Africa Consortium for Advanced Biostatistics (SSACAB) Consortium partners and the contact persons at each institution.

Institution	Principal Investigator	Principal Investigator Email
University of the Witwatersrand, Johannesburg (WITS)	Prof Tobias Chirwa	Tobias.Chirwa@wits.ac.za
Kilimanjaro Christian Medical College (KCMUCO)	Prof Michael Mahande	jmmahande@gmail.com
Stellenbosch University (SU)	Prof Taryn Young	tyoung@sun.ac.za
University of Namibia (UNAM)	Prof Lawrence Kazembe	lKazembe@unam.na
University of Zambia (UNZA)	Prof Patrick Musonda	pmuzho@hotmail.com
University of Nairobi (UoN)	Prof Patrick Weke	onyango@uonbi.ac.ke or pweke@uonbi.ac.ke
Makerere University (Mak)	Prof Nazarius Mboma Tumwesigye	naz@musph.ac.ug
Institut Supérieur des Techniques Médicales, Kinshasha (ISTM)	Prof Marie Claire	claireoma30@yahoo.com
University Medical Center Utrecht	Prof Marinus Eijkemans	M.J.C.Eijkemans@umcutrecht.nl
University of KwaZulu Natal (UKZN)	Prof Henry Mwambi	MwambiH@ukzn.ac.za
Kwame Nkrumah University of Science and Technology, Kumasi, Ghana (KNUST)	Dr. Emmanuel Kweku Nakua	enakua.chs@knust.edu.gh emmanngh@gmail.com
University of Malawi (UNIMA)	Dr Jupiter Simbeye	jsimbeye@cc.ac.mw
KEMRI Wellcome Trust Research Programme (KWTRP)	Prof Samson Kinyanjui	skmuchina@kemri-wellcome.org
South African Medical Research Council (SAMRC)	Prof Samuel Manda	Samuel.Manda@mrc.ac.za
Centre for the Aids Programme of Research in South Africa (CAPRISA)	Dr Nonhlanhla Yende- Zuma	nonhlanhla.yende@caprisa.org
London School of Hygiene & Tropical Medicine (LSHTM)	Prof Jim Todd	Jim.Todd@LSHTM.ac.uk
Northumbria University	Prof Ngianga-Bakwin Kandala	N-B.Kandala@warwick.ac.uk
Human Sciences Research Council (HSRC)	Prof Zuma Khangelani	kzuma@hsrc.ac.za

### Development, strengthening and implementation of biostatistics Masters level training

Each partner institution developed its curriculum for a Masters programme in biostatistics fitting within the local teaching capacity at each institution, and the regulations for Masters level training in each country. The Northern partners supported the development of specific modules and short courses that would benefit students and academic staff. SSACAB funds included support for administrative work on the program and fellowships for a total of 90 biostatistics students across the 11 institutions. SSACAB leadership reviewed the curriculum at each institution to ensure a basic minimum required for high-quality programmes in the region which could attract highly competent students. Further, to provide comparable standards across the courses, SSACAB aimed to work towards accreditation of the courses from the Royal Statistical Society of the UK.

### Providing PhD level training to develop expertise, skills, and become research leaders in biostatistics in Africa

The objective for building sustainable networks for biostatisticians envisaged close collaboration between academic training courses and the research institutions that undertake medical research. For high-quality cutting edge research questions and application of the advanced biostatistical methodology, co-supervision from members of both institutions was essential. This often starts with students planning a masters research project located within one of the research institutions. Such students can build a pipeline into PhD training, although to date it was mostly possible for fellows to join at the PhD level having trained elsewhere. 

### Building a sustainable network of biostatisticians and statistically informed researchers within each country through outreach, mentoring and transferring skills, workshops and conferences

**Figure 2.**
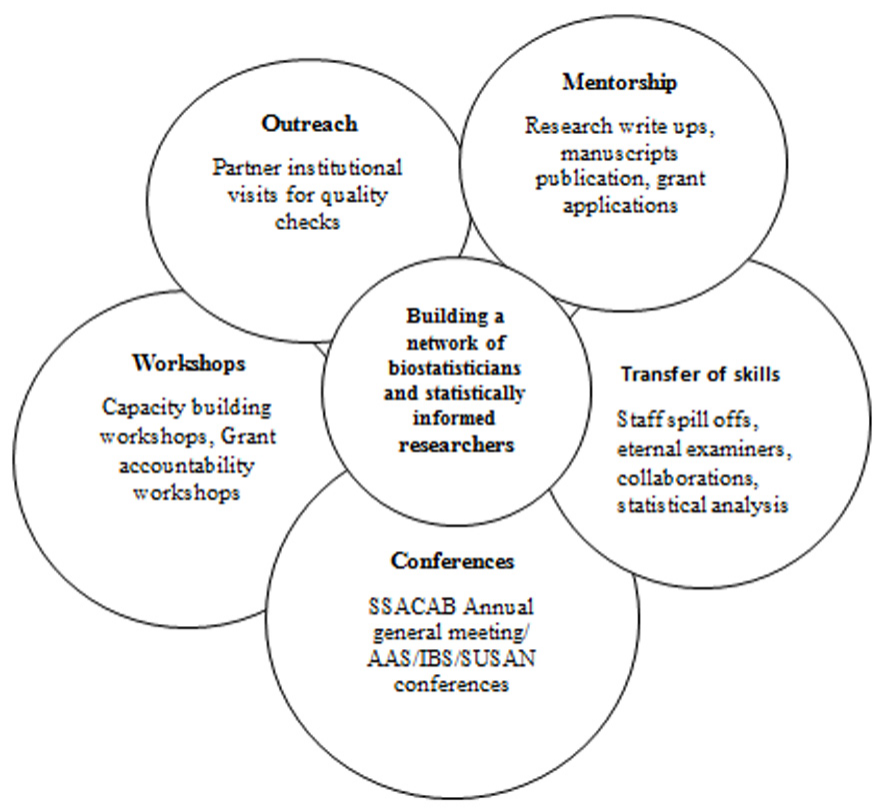
Illustration of the pillars underlying the Sub-Saharan Africa Consortium for Advanced Biostatistics (SSACAB) programme.

The third objective look towards the long-term impact of SSACAB based on the pillars illustrated in Figure 2. Integrating SSACAB meetings with national and regional statistical societies meetings allowed for greater synergy to encouraged biostatistics students and staff from SSACAB to take a role in the leadership and management of biostatistics societies. It also encouraged members to participate in meetings to present their work and network with colleagues. Apart from sharing biostatistical knowledge and scientifically sound research output, the networks provide a quality check for programmes, statistical analyses and manuscripts in preparation for submission. 

## Impact of SSACAB

To allow a smooth flow of the implementation and coordination of the SSACAB programme, one or two representative institutions from a given country based on evidence of biostatistics research and training were selected to join the programme. However, candidates to benefit from the SSACAB could come from any institution and country across the SSA region. Of the 11 African universities partnering in SSACAB, four had developed a Masters level program in Biostatistics before the start of SSACAB in 2015 ( Table 2). Within five years of SSACAB’s existence, all remaining universities have developed and started teaching Masters in Biostatistics programmes with assistance from SSACAB.

**Table 2.** Sub-Saharan African Consortium for Advanced Biostatistics (SSACAB) partner institutions and their corresponding Biostatistics programme set-up details.

Partner	Country	Year Masters Biostatistics started	Department/Division/Unit Masters is situated	Statistics staff size ^1, 2^	External collaboration on teaching ^2^
University of the Witwatersrand, Johannesburg (WITS)	South Africa	2016	School of Public Health, Division of Epidemiology and Biostatistics	4	UMCU, Northumbria, SAMRC, HSRC
Kilimanjaro Christian Medical College (KCMUCO)	Tanzania	2010	Institute of Public Health, Department of Epidemiology and Biostatistics	3	LSHTM, UKZN, SAMRC, WITS,
Stellenbosch University (SU)	South Africa	2017	Division of Epidemiology and Biostatistics, Department of Global Health	9	LSHTM, McMaster University, UNZA, UKZN, Hasselt University, SAMRC, UCT, WITS
University of Namibia (UNAM)	Namibia	2017	Department of Statistics and Population Studies, Statistics Unit	7	WITS
University of Zambia (UNZA)	Zambia	2016	School of Public Health, Department of Epidemiology and Biostatistics	6	LSHTM
University of Nairobi (UoN)	Kenya	2000	School of Mathematics, School of Mathematics	20	Hasselt University, LSHTM
Makerere University (Mak)	Uganda	2018	School of Public Health, Epidemiology and Biostatistics	-----	-----
Institut Supérieur des Techniques Médicales, Kinshasha (ISTM)	Democratic Republic of Congo	2019	ISTM (Doctoral school), Biostatistics	4	University of Nairobi, WITS, Northumbria
University of Kwa-Zulu Natal (UKZN)	South Africa	Existed before the inception of SSACAB programme	School of Mathematics, Statistics and Computer Science, Statistics	17	Hasselt University, KCMUCO, Harvard Biostatistics Department, Stellenbosch University, WITS, LSHTM, University of South Carolina, Ghent University, SAMRC, UCT, HSRC.
University of Malawi (UNIMA)	Malawi	2010	Mathematical Sciences Department	5	MLW, MZUNI, UNAM, WITS, SAMRC
Kwame Nkrumah University of Science and Technology, Kumasi, Ghana (KNUST)	Ghana	2019	Department of Epidemiology and Biostatostics	4	LSHTM

1. Staff trained to Masters or PhD in biostatistics (including Honorary positions), 2. As of September 2019. HSRC= Human Sciences Research Council; ISTM= Institut Superieur Technique Medical; KCMUCo= Kilimanjaro Christian Medical University College; KNUST= Kwame Nkrumah University of Science and Technology; LSHTM=London School of Hygiene and Tropical Medicine; MLW=Malawi-Liverpool Wellcome Trust, College of Medicine, University of Malawi; MZUNI=Mzuzu University; SAMRC =South African Medical Research Council; UCT= University of Cape Town; UKZN=University of KwaZulu Natal; UMCU = University Medical Center Utrecht; UNAM= University of Namibia; UNZA= University of Zambia; WITS= University of the Witwatersrand

The University of the Witwatersrand gained Royal Statistical Society accreditation in 2019, the first African university to do so. The development of all programmes has involved national accreditation and higher education institutions approval for Masters level training, which requires a commitment from the university for the appointment of lecturers and professors with appropriate biostatistics qualifications and expertise. SSACAB has also enabled external support to the programs in developing Masters level modules like infectious disease modelling, Bayesian modelling and spatial modelling which are universal across all the institutions; and provides assistance to teach these modules. This involved Northern partners from SSACAB as well as from within other SSACAB institutions, who teach alongside the local faculty staff at institutions to build the institutional capacity to develop and deliver new courses and modules.

**Figure 3.**
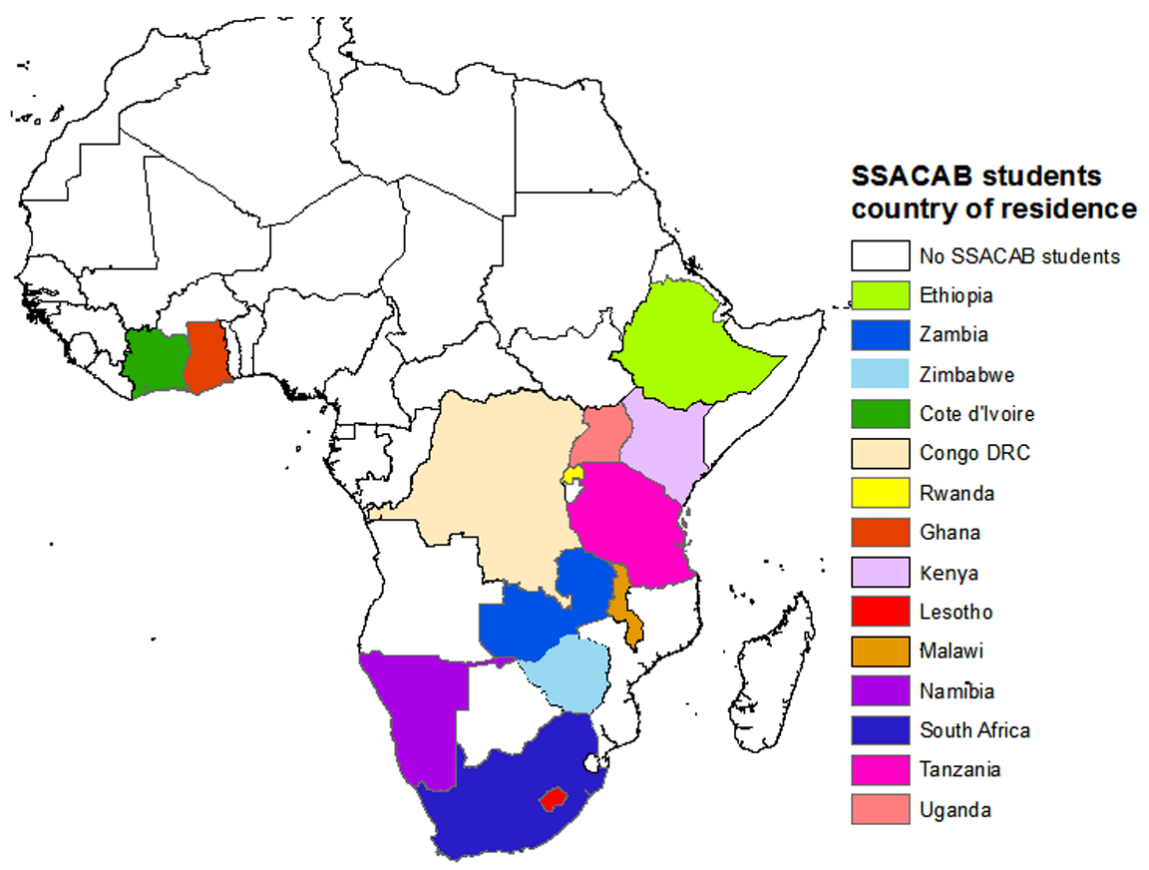
Sub-Saharan Africa Consortium for Advanced Biostatistics (SSACAB) fellows and their institutions.

A total of 150 fellows have been awarded scholarships to date from 14 different countries in SSA ( Figure 3). Since the inception of the SSACAB in 2016, a total of 123 Masters have been awarded a fellowship as of 2019 ( Table 3).

**Table 3.** Masters in Science (Masters) and Doctoral (PhD) degrees in biostatistics enrolments, with number supported by the Sub-Saharan African Consortium for Advanced Biostatistics (SSACAB) in brackets ().

Degree programme	Masters degree				PhD degree			
**Year** **Partner**	2016	2017	2018	2019	2016	2017	2018	2019
University of the Witwatersrand, Johannesburg (WITS)	(4)	(4)	(7)	(6)	(1)	(1)	(2)	(3)
Kilimanjaro Christian Medical University College (KCMUCO)	10 (1)	5(2)	10(6)	10 (3)	-----	2	2 (1)	-----
Stellenbosch University (SU)	-----	5(5)	(5)	8(2)	-----	(1)	1 (1)	-----
University of Namibia (UNAM)	-----	(3)	(2)	(1)	------	-----	(1)	(1)
University of Zambia (UNZA)	(3)	(2)	------	(3)	4	-----	------	-----
University of Nairobi (UoN)	(1)	(3)	(6)	-----	-----	2 (1)	2 (2)	2
Makerere University (Mak)	-----	-----	15(10)	13(10)	-----	-----	-----	-----
Institut Superieur Technique Medical, Kinshasa (ISTM)	-----	-----	-----	(7)	-----	-----	-----	-----
University of Kwa-Zulu Natal (UKZN)	(2)	(4)	(4)	(4)	(3)	(1)	(2)	(2)
University of Malawi (UNIMA )	10 (4)	15 (3)	10 (3)	10 (3)	-----	1 (1)	-----	(1)
KEMRI Welcome Trust Research Programme (KWTRP)	-----	-----	-----	-----	-----	(1)	(1)	-----
Kwame Nkrumah University of Science and Technology, Kumasi, Ghana (KNUST)								
**Total (SSACAB fellows only)**	**35 (15)**	**51(26)**	**78(43)**	**80(39)**	**4(4)**	**11(6)**	**15 (10)**	**9(7)**

The Masters students in the 11 SSACAB partner institutions are taught over a range of modules shown in Table 4 with the teaching models varying by institutions. Most Masters courses have an initial biostatistical foundation course, which is also available to Masters students following other disciplines, including Medical Officers on residency for MMED degrees ( Table 4). This facilitates new ways to enhance the basic statistical applications available to medical doctors and other health professionals in their studies. In general, a Masters student takes theoretical modules which are taught in class, conducts a research project as part of a research internship and engages in statistical consultancy before graduating within two years.

**Table 4.** Outline and, duration of teaching modules for Sub-Saharan African Consortium for Advanced Biostatistics (SSACAB) partner institutions.

Institutions	WITS (1)	KCMUCO (2)	SU (3)	UNAM (4)	UNZA (5)	UoN (6)	Mak (7)	ISTM (8)	UZKN (9) #	UNIMA (10)	KNUST (11)
Foundation of Maths & Statistics	3	2			2 *		0.5 *		1		
Inference	2		2 *	2	2 *	1.5 *	1 *+1 *	2	1	1	1
Statistics and probability theory	2 *		2 *	2	2 *				1	1+1+1	
Surveys, Study & research design	1	2+1		2	1 *+1 *	1.5 *+1.5 *	0.5 *+0.5 *	2+2	1	1	1
Generalised Linear Models	2	2+1+1	2 *+2 *	2	2+1 *+1 *	1.5 *+1.5 *	3 *	2	1	1	1+1
Survival & time to event		2	2 *	2	2+2	1.5 *	2 *	2	1	1	2
Epidemiology	1	2+4	2 *	2	2+2		1 *+1 *		-----	1	1+1+1+1
Clinical Trials	1		2 *	2		1.5 *		2	1+2	1	
Analysis and Modelling	1	2			2	1.5 *+1.5 *	1 *+1 *	2	-----	1+1+1	1
Multilevel models		2 *	2 *	2	2		2 *	2	0.5	1+1	2
Spatial	2								0.5	1	1
SEM and Causal modelling			2 *		2 *		1 *		-----		
Bayesian Analysis	1		2 *		2 *		1 *	2	1+2	1	
Database, computing and data management	1 *+1 *	2	2 *			1.5 *	1 *+1 *+1 *		1+2		1
Other courses (1)	2	2+1+1			1 *	1.5 *+2		2+2+1+1		1	1+1+1+1
Professional Attachment			12		8				-----		3
Research Project	26	14	26				10	24	104		20
Taught course time (weeks)	19	27	20	16	28	18.5	24.5	18	-----	18	18

Notes: Numbers indicate the number of weeks of teaching, excluding assignments, exams and student work; # UKZN Masters by research, taught modules are all optional; * Blended over weeks (approx weekly equivalent); + indicates more than one course on the topic over the Masters; [1] WITS=University of the Witwatersrand, South Africa; [2] KCMUCO=Kilimanjaro Christian Medical University College, Tanzania; [3] SU=Stellenbosch University, South Africa; [4] UNAM= University of Namibia; [5] UNZA= University of Zambia; [6]UoN= University of Nairobi; [7] Mak=Makerere University, Uganda; [8] ISTM=Institute Suprereur Technique Medical, Democratic Republic of Congo; [9] UKZN=University of KwaZulu Natal, South Africa ;[10] (UNIMA)=University of Malawi; [11] KNUST= ****Kwame Nkurumah University of Science and Technology, Ghana. Other courses include Statistical Consulting, Professional issues, Time series, Monitoring and Evaluation, English language, Communication, Qualitative Analysis, Psychology, Leadership, Teaching, Laboratory data, Agricultural Experiments.

The value of the Masters programmes is reflected in the further professional development of the students following graduation and the outputs from their studies ( Table 5). Of the 123 Masters students enrolled, 41 students have graduated in 2019, 19 students have been employed in African academic institutions while 15 students are working in African research institutions. Thirteen Masters graduates have been enrolled in PhD programmes. Two of students who have graduated have been employed in the government ministries. Although the number is currently low, the programme is expected to contribute to government institutions with time. To date, 17 Masters students have been able to publish their research in a peer-reviewed journal.

**Table 5.** Sub-Saharan African Consortium for Advanced Biostatistics (SSACAB) student achievements: Graduated, Employed, Publications & Subsequent career development as of December 2019 in any African institution.

Partner Institution	Number of Masters students Enrolled	Number of Masters students Graduated	Number of graduates employed in an academic institution	Number of graduates enrolled in PhD	Number of graduates working in the Ministry of Health	Number of graduates working in Research Institutions	Number of Masters Publications
University of the Witwatersrand, Johannesburg (WITS)	(21)	(8)	(0)	(2)	(0)	(0)	(2)
Kilimanjaro Christian Medical University College (KCMUCO)	35 (12)	(9)	(7)	(1)	(2)	(5)	(3)
Stellenbosch University (SU)	13(12)	(5)	(1)	(1)	(0)	(2)	(1)
University of Namibia (UNAM)	(6)	------	------	------	------	------	------
University of Zambia (UNZA)	(8)	(1)	(0)	(0)	(0)	------	(2)
University of Nairobi (UoN)	(10)	(4)	(3)	(3)	(0)	(3)	(2)
Makerere University (Mak)	23 (20)	------	------	------	------	------	------
Institut Superieur Technique Medical, Kinshasa (ISTM)	(7)	------	------	------	------	------	------
University of Kwa-Zulu Natal (UKZN)	(14)	(8)	(2)	(3)	(0)	(2)	(6)
University of Malawi (UNIMA)	45 (13)	(6)	(6)	(3)	(0)	(3)	(1)
Kwame Nkrumah University of Science and Technology, Kumasi, Ghana (KNUST)							
**Total**	**239(123)**	**(41)**	**(19)**	**(13)**	**(2)**	**(15)**	**(17)**

Initially, the SSACAB had planned to award fellowships to 15 PhD students; with support from several co-funders by the end of 2019, a total of 27 (10 of which were female) PhD students have been awarded fellowships ( Table 3). The data (SEARCH) project has supported an additional two PhD students at the Kilimanjaro Christian Medical College (KCMUCO) and four at the University of Zambia (UNZA), while a capacity-building grant from Glaxo Smith Kline (GSK) has supported a further six PhD students (two at the University of Witwatersrand (WITS), two at the University of KwaZulu Natal (UKZN), one at Stellenbosch University (SU) and one at the University of Nairobi (UoN). Additional funding has been provided by the German Academic Exchange Service (DAAD) for Masters fellowships in biostatistics. Additionally, other research projects have paid for their staff to be trained at SSACAB supported institutions having seen the success of the programme.

Other Wellcome Trust DELTAS consortia have supported several Masters students within SSACAB with joint funding of the KCMUCO programme from Training Health Researchers into Vocational Excellence in East Africa (THRiVE). The co-funding from other projects enhances the integrative supervision for most of the PhD students as staff from the Northern universities co-supervise the PhD students. Training institutions have forged collaborations with research institutions within SSACAB to support joint supervision of Masters and PhDs, external examination of courses and research reports as well as sharing curricula, thereby increasing the visibility of the consortium and improving quality of the Masters and PhD programmes. Four of the PhD students are due to graduate by 2020. There are currently 31 peer-reviewed publications from PhD fellows and a further two from staff supported by SSACAB ( Table 6).

**Table 6.** Details of PhD students enrolled at Sub-Saharan African Consortium for Advanced Biostatistics (SSACAB) institutions and their publication outcomes.

Partner Institution	Number of PhD enrolled to date	Grant	Supervisors’ affiliations	Publications To date
University of the Witwatersrand, Johannesburg (WITS)	(7)	4- SSACAB 3-GSK	WITS, LSHTM, UMCU	5
Kilimanjaro Christian Medical University College (KCMUCO)	(1)	SSACAB	KCMUCO, LSHTM and UKZN	1
Stellenbosch University (SU)	(2)	1- SSACAB 1-GSK	SU, LSHTM	1
University of Namibia (UNAM)	(2)	2- SSACAB	UNAM	1
University of Nairobi (UoN)	(3)	2- SSACAB 1-GSK	UoN, LSHTM, and UMCU	2
University of Kwa-Zulu Natal (UKZN)	(8)	5- SSACAB 2-GSK	UKZN, USA, KCMUCO, SAMRC	18
University of Malawi (UNIMA )	(2)	1- SSACAB	UNIMA	1
KEMRI Welcome Trust Research Programme (KWTRP)	(2)	SSACAB	KEMRI Welcome Trust Research Programme (KWTRP)	2
**Total**	**(27)**			**31**

*SSACAB= Sub-Saharan African Consortium for Advanced Biostatistics; GSK= Glaxo Smith Kline; LSHTM=London School of Hygiene and Tropical Medicine; USA=United States of America; SAMRC=South Africa Medical Research Council; WITS=University of the Witwatersrand, South Africa; KCMUCO= Kilimanjaro Christian Medical University College; SU=Stellenbosch University; UoN= University of Nairobi; UNAM= University of Namibia; UKZN=University of KwaZulu Natal, South Africa; UNIMA=University of Malawi; KEMRI=Kenya Medical Research Institute, KWTRP= KEMRI Welcome Trust Research Programme, UMCU = University Medical Center Utrecht

SSACAB has also partially supported other PhD students enrolled in partner institutions with their manuscript publication fees in peer-reviewed open journals. Furthermore, staff members within SSACAB have also been supported in publishing their research work and in presenting their work at international conferences. This partial support of staff and student research has resulted in approximately 10 publications. Some staff supported research have resulted in the publication of books, including the ‘Statistical Modelling of Complex Correlated and Clustered Data Household Surveys in Africa’ edited volume from the University of Namibia ( Ngianga-Bakwin & Lawrence, 2019). 

SSACAB has encouraged and supported networking of students and consortium members through participation at national and international conferences; travel grants have often been provided to assist their attendance. Notable conferences include the Statistics Association of South Africa (SASA) conference, and the Statistics Conference organised by the University of Malawi in partnership with Statistical Association of Malawi, SSACAB Annual Research Conferences, SSACAB Annual General Meetings, sub-Saharan Africa Network (SUSAN)- SSACAB conferences and the International Biometrics Society (IBS) conference in Uganda. The SSACAB programme managed to work with the IBS which led to the accreditation of Masters programmes by IBS and integration of meetings including the first-ever Joint conference of the IBS/ SSACAB held in 2019. Such meetings integration with IBS have been done to support quality education and state of the art biostatistical methodology within SSACAB. Local research institutes within SSACAB have supported students through hosting students during their research period. Masters and PhD students have been supported with data generated by these research institutes for their dissertation and thesis reports. Moreover, the students have also had the opportunity to interact with research experts in other public health fields. Collaboration between academic universities and the local research institutions enable joint supervision of students, providing greater insight into the statistical issues that need to be considered when handling research data.

## Discussion

The SSACAB’s ultimate goal was to create a research node of excellence (scientific citizenship) through contributions to science, policy and practice; growing the biostatistics discipline and nurture upcoming researchers with advanced skills and expertise (research training). The SSACAB goals are well intertwined within the DELTAS Africa strategic areas in health. The SSACAB programme initiative specifically benefits the African continent in terms of expanding the biostatistical capacity based on the needs identified by African institutions.

The SSACAB initiative came at a time when the global focus has shifted towards novel data analysis concepts, including big data analysis to support evidence-based health sciences. Over time, the SSACAB programme has accommodated these new methods through advanced biostatistics teaching modules addressing big data analysis challenges and by increasing the number of partner institutions that can provide biostatistical training. This is a positive stride towards achieving greater coverage of biostatisticians across Africa and in widening the scope for new biostatisticians working on cutting edge analyses. At the inception of the programme, only four institutions had an established Masters programme in biostatistics. Through the SSACAB, a professional research environment has been provided to institutions to facilitate their biostatistical research and ensure high-quality post-graduate degree training. This SSACAB support has ensured that biostatistics researchers are given adequate resources and mentorship to develop their interests in statistical concepts and methods.

The SSACAB consortium has achieved its research training goals as many Masters and PhD students have been awarded fellowships andthe timely graduation of these students for both Masters and PhD programmes. The research training has allowed the fellows to strengthen their professional development and provides a career pathway through progression to PhD level and postdoctoral training; and employment in leading academic and research institutions. Several of these trained statisticians are now working within various entities in the Ministry of Health, other government agencies and non-governmental organisations (NGOs) in different SSA countries. Such involvement of biostatisticians in government structures supports evidence-based policy and planning. Mentorship and building of biostatistics research leadership are important to consolidate this initial impact further and make a lasting contribution to health research in SSA. The diversity of courses offered, ranging from theoretical statistics to applied epidemiology could be viewed as one of the strengths of the SSACAB consortium since this provides a wide diversity of career options to students to choose from.

Scientific citizenship in the African context has been facilitated in several ways. Firstly, the SSACAB Masters and PhD fellows across all the 11 partner institutions have to date produced 44 peer-reviewed scholarly publications addressing major health issues in SSA. These publications are a measure of how upcoming researchers are nurtured in the biostatistics programme to produce high-quality research to inform policy and practice in the African context and beyond. Secondly, fellows shared these research outputs through other platforms to ensure that the research findings are disseminated to peers and policymakers in Africa. At the same time, public engagement and awareness have been supported through press, social media platforms and community activities to increase the uptake of new health research findings regionally and beyond. In other words, the impact of SSACAB has not been limited to offering awards to post-graduates but has been felt in the generation of high-quality research to inform policy in SSA. There were research presentations at the AAS Conference in Senegal and female genital mutilation (FGM) work presented to the United Nations. Globally, SSACAB work has been presented at the 61st Session of the World Congress of the International Statistical Institute (ISI) in Morocco and the 62nd ISI World Statistics Congress 2019 (ISIWSC 2019) in Malaysia. 

Collaborative research supervision through South-South and North-South partnership is an important component of the success and has significantly impacted on the quality of teaching and research supervision. Senior statisticians from well-resourced universities have assisted with teaching courses and supervision of graduate research projects. Research institutions have supported research internship and attachments through the provision of data generated in these research institutions. This has strengthened the research capacity of the students and opens up more opportunities for upcoming biostatisticians as they would interact with senior researchers and experts in various public health fields ( Bates *et al.*, 2006). These internships and attachments have also provided an opportunity for fellows to engage with health research studies of direct policy significance. This is an important stride accomplished by SSACAB as a future generation of researchers is being shaped to become professionals who will take part in shaping and driving the locally relevant health research agenda which will contribute to the improvement and development of health in Africa ( Thabane *et al.*, 2008).

Another crucial highlight within the SSACAB programme has been the spin-off of academic appointments at collaborating institutions as well as the involvement of staff from various institutions as external examiners for structured modules and research reports in other institutions. Such inter-institutional collaboration and involvement of staff opened a platform of content sharing and networking; hence, strengthening the biostatistics programmes at the same time maintaining the quality of the deliverables. Not only has biostatistics been offered to post-graduate students (Masters and PhD) but there has been a promotion of biostatistics to undergraduate students by seasoned researchers within the consortium through research mentorship and consultations.

Most of the substantial achievement of the SSACAB has been attained with modest financial means. The partner institutions pooled limited resources for joint teaching, which resulted in the development of advanced modules being taught in Masters programmes. Many institutions have also obtained funding from other sources which resulted in additional enrolment of students while maintaining the quality of the students’ output at these institutions. These initiatives lay the foundation for the long-term sustainability of the programme to run beyond the official funding phase of SSACAB.

## SSACAB challenges

SSACAB challenges included different criteria for Masters students’ enrolment and fellowship awards. This approach might have contributed to the delayed graduation of some Masters fellows. The variations in the quality of undergraduate statistics training feeding into the Masters programme could have affected the quality of Masters graduates. This same challenge was also observed in the Masters students enrolled for PhD. In instances where the Masters students lacked fundamental theory, the PhD students were encouraged to take Masters courses offered at their host institutions. The shortage of academic teaching staff has hindered smooth delivery of teaching at some institutions. While visiting staff would relieve the burden and provide new teaching perspectives; however, this is more expensive compared to having permanent academic staff. This is one of the challenges that SSACAB graduates can help to remedy. Timely financial reporting has been a challenge in some institutions, which lack the experienced staff to administer and report funds. To familiarise financial managers with the reporting structures and grant conditions for the Alliance for Accelerating Excellence in Science in Africa (AESA)/Wellcome Trust, SSACAB facilitated a workshop in South Africa in 2017 and Nairobi, Kenya in 2018. This realigned the SSACAB programme to meet the expectations of researchers, partners and funders for future collaborative activities. 

## Conclusion

In the five years, the SSACAB has made tremendous progress in the growth of biostatistics capacity and resources in the SSA region. Now, more than ever there is great awareness and uptake of local biostatisticians into health progress, in addition to an increase in peer-reviewed publications by biostatisticians in the region. The programme has nurtured upcoming researchers with advanced skills and expertise, and create a research node of excellence. Significant strides have been made for each aspect: the enrolment of fellows has surpassed expectation, the Masters programmes are becoming recognised for their excellence, and professional biostatistical networks are flourishing. These achievements need to be consolidated with a career pathway for biostatisticians and data professionals within the health research community.

## Data availability

### Underlying data

No data are associated with this article
