# Supplementary material for: Developing excellence in biostatistics leadership, training and science in Africa: How the Sub-Saharan Africa Consortium for Advanced Biostatistics (SSACAB) training unites expertise to deliver excellence
Source: AAS Open Res. 2020 Dec 22;3:51. Originally published 2020 Oct 5. [Version 2] doi: 10.12688/aasopenres.13144.2 (PMC7802118; doi:10.12688/aasopenres.13144.2)
Supplement: Peer review history

# Peer review: Reviewer response for version 2

**DOI:** 10.21956/aasopenres.14290.r27990

**Published:** 2021-01-11

**Authors:** Sylvia Kiwuwa-Muyingo

**Affiliations:** 1 African Population and Health Research Center, Nairobi, Kenya

The rationale for the open letter is very clear and addresses a critical need to enhance biostatistical capacity on the African continent. The Sub-Saharan African consortium for advanced biostatistics aim was to develop capacity of masters level training in biostatistics; secondly provide  expertise skill to becoming a research leader in biostatistics at PhD level through collaborations between academic and research institutions; and build a network of biostatisticians within each country.

**Does the article adequately reference differing views and opinions?**

 While the article clearly addresses a challenge that is very pertinent to demand for biostatisticians in the region, I think it might benefit from  addressing a broader view of the career choices for students that have since transitioned to be employed in academia or research institutions and beyond, say mentorship on what to do when the PhD is finished? It is clear most students would like an academic job, which is not easy in the first few years but preferably in the University where they studied so they might opt for a postdoc/position to consolidate their research but there are few flexible postdoc positions.

 In fact the lack of awareness of career pathways for biostatisticians might imply many opt to remain in academia or research institutions. There are a lot more flexible opportunities for the semi-academic route such as service statisticians in public health organisations to provide statistical support to other disciplines. Another is the business route for the growing number in consulting, industry, financial institutions. The downside of these organizations is the lack of independence and bureaucracy. The discussion about other possibilities and the fellows awareness in those areas might be helpful to the reader.

**Where applicable, are recommendations and next steps explained clearly for others to follow?**

 Beyond integration of SSACAB within existing statistical organisations is the need to recognize that society operates in a nested system in which individuals exist within communities which then contribute to national and regional entities. This plan could highlight the interaction with different actors at each or high level including social, economic and policy actors to effect policy change or practice beyond integration with existing statistical organisations.

 Another key challenge for many researchers especially that might be addressed in future plans for biostatistics is the need for "research communication" - a critical skill for communicating research findings to the public and for seeking funding. While publications are top of the list, a lot more is required to communicate good science. It is not very clear from the article if/how communication - very essential for fellows - contributes to informing policy and practice .

Does the article adequately reference differing views and opinions?

Partly

Are all factual statements correct, and are statements and arguments made adequately supported by citations?

Yes

Is the Open Letter written in accessible language?

Yes

Where applicable, are recommendations and next steps explained clearly for others to follow?

Partly

Is the rationale for the Open Letter provided in sufficient detail?

Yes

Reviewer Expertise:

Biostatistics, HIV and Mental health, Adherence and Women's health,

I confirm that I have read this submission and believe that I have an appropriate level of expertise to confirm that it is of an acceptable scientific standard.

# Peer review: Reviewer response for version 2

**DOI:** 10.21956/aasopenres.14290.r28246

**Published:** 2021-01-05

**Authors:** Victoria N. Nyaga

**Affiliations:** 1 Unit of Cancer Epidemiology, Belgian Cancer Centre, SCIENSANO, Brussels, Belgium

No further comments to make.

Does the article adequately reference differing views and opinions?

Partly

Are all factual statements correct, and are statements and arguments made adequately supported by citations?

Yes

Is the Open Letter written in accessible language?

Partly

Where applicable, are recommendations and next steps explained clearly for others to follow?

Not applicable

Is the rationale for the Open Letter provided in sufficient detail?

Partly

Reviewer Expertise:

Biostatistics

I confirm that I have read this submission and believe that I have an appropriate level of expertise to confirm that it is of an acceptable scientific standard.

# Peer review: Reviewer response for version 1

**DOI:** 10.21956/aasopenres.14245.r27977

**Published:** 2020-11-03

**Authors:** Michael G. Chipeta

**Affiliations:** 1 University of Oxford, Oxford, UK

The open letter describes SSACAB’s initiatives to nurture biostatistics skills in African biomedical scientists. Through the initiative, a number of things have been achieved including the provision of professional research environments at participating centers, development of post-graduate training (MSc and PhDs) as well as high-quality publications.

 The letter addresses the development, strengthening, and implementation of both MSc and Ph.D. levels in biostatistics. What is conspicuously missing, and therefore needs to be included, is a statement on the quality of undergraduate training in biostatistics/statistics that feeds into MSc training and subsequently, into Ph.D. training. The success in the development of biostatistics research excellence (at MSc/Ph.D.), in part and largely, depends on a solid foundation at the BSc level. A statement on the quality of undergraduate biostatistics/statistics training at the various participating institutions will be useful in highlighting the successes and/or challenges in attracting talent to the biostatics profession as well as the sustainability of the initiative. Additionally, the authors need to comment on efforts made, if any, to support the development of statistical training curricula at the undergraduate level.  

 The letter should include a section dedicated to the challenges that have been faced in the initiative to develop and nurture biostatistics. Future initiatives would build on addressing these alongside strengthening the existing successes described in the letter.

Does the article adequately reference differing views and opinions?

Partly

Are all factual statements correct, and are statements and arguments made adequately supported by citations?

Yes

Is the Open Letter written in accessible language?

Yes

Where applicable, are recommendations and next steps explained clearly for others to follow?

Partly

Is the rationale for the Open Letter provided in sufficient detail?

Yes

Reviewer Expertise:

Biostatistics

I confirm that I have read this submission and believe that I have an appropriate level of expertise to confirm that it is of an acceptable scientific standard, however I have significant reservations, as outlined above.

# Response

**Published:** 2020-11-30

**Authors:** Zvifadzo Matsena Zingoni

**Affiliations:** Witwatersrand University, South Africa

Thank you very much for having some time to review our manuscript. We have noted the raised comments and we have included the information in the revised version of our manuscript.

# Peer review: Reviewer response for version 1

**DOI:** 10.21956/aasopenres.14245.r28029

**Published:** 2020-10-23

**Authors:** Victoria N. Nyaga

**Affiliations:** 1 Unit of Cancer Epidemiology, Belgian Cancer Centre, SCIENSANO, Brussels, Belgium

- I think there should a section dedicated to challenges faced as opposed to a paragraph on challenges faced in the discussion.
- There are existing initiatives like the sandwich PhD programme by vlirous.  Why doesn't SSACAB partner with VLIROUS while they also have the same goal as SSACAB? How do such initiatives fail or how is SSACAB better?
- There are a couple of incomplete sentences, or sentences that need rephrasing. 
- Please see the PDF file here for additional comments.

Does the article adequately reference differing views and opinions?

Partly

Are all factual statements correct, and are statements and arguments made adequately supported by citations?

Yes

Is the Open Letter written in accessible language?

Partly

Where applicable, are recommendations and next steps explained clearly for others to follow?

Not applicable

Is the rationale for the Open Letter provided in sufficient detail?

Partly

Reviewer Expertise:

Biostatistics

I confirm that I have read this submission and believe that I have an appropriate level of expertise to confirm that it is of an acceptable scientific standard, however I have significant reservations, as outlined above.

# Response

**Published:** 2020-11-30

**Authors:** Zvifadzo Matsena Zingoni

**Affiliations:** Witwatersrand University, South Africa

Thank you very much for having time to review our manuscript. We have revised our manuscript faithfully following your suggestions. Below are some responses to some of the questions raised:

***I think there should be a section dedicated to challenges faced as opposed to a paragraph on challenges faced in the discussion.***

 Thank you for the comment. We have made this a stand-alone section in the discussion as suggested.

***Why is the vlirous not in the list of northern partners?*** ***There are existing initiatives like the sandwich PhD programme by vlirous.  Why doesn't SSACAB partner with VLIROUS while they also have the same goal as SSACAB? How do such initiatives fail or how is SSACAB better?***

 Thank you for introducing Vlir-ous to us, an organisation supporting partnerships between universities and university colleges, in Flanders (Belgium) and low- and middle-income countries. SSACAB, funded by the Wellcome Trust under the Trust’s ‘Developing Excellence in Leadership, Training and Science’ has a strong focus on the development of Africa-led networks for science research in SSA and the development of South-South partnerships. As such, SSACAB tries as much as possible to have collaborations with similar initiatives based in the SSA region. However, we have several SACCAB scientists working with various universities in Belgium that have students under Vlir-ous., which has resulted in some workshops organised in SACCSAB sponsored meetings and conferences. Moreover, the unique feature about SSACAB is that it is a locally initiated and implemented consortium with northern institutions joining as partners and collaborators. The impact of SSACAB in SSA is that is has a great potential to successfully retain trained MSc, PhD and Post-Doc Biostatisticians within the region which is normally less likely if fellows are trained outside Africa.

***Are all these co-authors really needed to review and edit? If so, why do you still need other reviewers (like me)?***

 Thank you for the comment. Co-authors have all contributed to the development and conduct of the SSACAB programme and the manuscript; peer-review is a very different process to ensure the external (methodological) quality of a paper.

***Why only 11? Or mention the selection criteria of the participating universities. EG, why would JKUAT from Kenya not be on the list?***

 SSACAB is one program to improve biostatistics in sub-Saharan Africa. It is not intended to include all MSc programs or all PhD training in SSA. SSACAB is not a regional network of biostatistical institutions, or biostatisticians. SSACAB is encouraging individuals and institutions to network through the national statistical associations and international institutions such as RSS and IBS. The aim of SSACAB is not to become a network of all institutions but to strengthen institutions to belong to a sustainable network such as IBS or RSS. In other words, SSACAB is highlighting the need to come together and strengthen networks of biostatisticians such as the SUSAN network under the International Biostatistics Society. JKUAT would be welcome to join and work with such national and international networks.

***There are a couple of incomplete sentences, or sentences that need rephrasing. Please see the PDF file here*** ***for additional comments.***

 Thank you for the comment. We have addressed all the comments accordingly.

***Only 2 graduates are in the govt?!, I am doubting their level and impact of involvement in the policy-making and planning.***

 Thank you for the comment. Although the number of SSACAB graduates working in government to date is low, we anticipate a significant impact these biostatisticians will have in the government institutions with time.
